# A Facile and Convenient Synthesis of some Novel Hydrazones, Schiff’s Base and Pyrazoles Incorporating Thieno[2,3-*b*]thiophenes

**DOI:** 10.3390/ijms12117824

**Published:** 2011-11-11

**Authors:** Yahia Nasser Mabkhot, Assem Barakat, Abdullah Mohammed Al-Majid, Zeid Abdullah Al-Othman, Abdullah Saleh Alamary

**Affiliations:** Department of Chemistry, Faculty of Science, King Saud University, P.O. Box 2455, Riyadh 11451, Saudi Arabia; E-Mails: amajid@ksu.edu.sa (A.M.A.-M.); zaothman@ksu.edu.sa (Z.A.A.-O.); alamary1401@yahoo.com (A.S.A.)

**Keywords:** bicyclic compounds, hydrazone, thieno[2,3-*b*]thiophenes, heterocycles, schiff’s base

## Abstract

A facile and convenient synthesis of some novel hydrazones, schiff’s base and pyrazoles from thieno[2,3-*b*]thiophene derivatives **1** have been achieved in high yields assisted by microwave and classical methods. The structures of all the title compounds have been elucidated by elemental analysis, IR, MS, ^1^H-NMR and ^13^C-NMR. Generally, these findings represent a new class of sulfur and nitrogen moieties that should also be of interest as new materials.

## 1. Introduction

Substituted thieno[2,3-*b*]thiophenes [1] have been extensively investigated for their biological or physical properties. The interest in this kind of heterocycles has spread from early dye chemistry [2] to modern drug design, [3] biodiagnostics, [4] electronic and optoelectronic devices, [5] conductivity-based sensors, [6] and self-assembled superstructures [7]. Thienothiophenes derivatives are valuable building blocks in synthetic organic chemistry [8–10]. Mabkhot and others [11–20] have reported a variety of syntheses of heteroaromatics developed using functionally substituted thieno[2,3-*b*]thiophenes as readily obtainable building blocks possessing multiple electrophilic and nucleophilic moieties. We decided to take advantage of our recent results in the synthesis of bis-heterocycles thieno[2,3-*b*]thiophenes derivatives [21,22], to extend the synthesis of a variety of schiff’s base and hydrazones bis-heterocycles systems for biological and pharmacological evaluation.

## 2. Results and Discussion

**Scheme 1:**
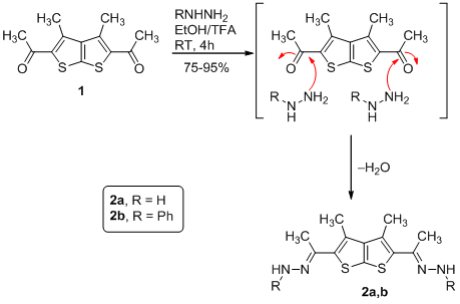
Synthesis of hydrazones derivatives **2a,b** from thienothiophene **1**.

We chose to combine the *N*-terminal and central portions of analogues first. Construction of the substituted thienothiophenes **2a,b** proceeded from initial condensation of the [2,3-*b*]thienothiophene central constraints with hydrazine derivatives. An example of our initial synthetic approach is outlined in Scheme 1.

[2,3-*b*]thienothiophene hydrazine **2a** was prepared from **1** with *N*-nucleophile such as hydrazine in EtOH under reflux for 4h in the presence of catalytic amount of TFA (trifluoro acetic acid) afforded **2a** and the condensation proceeded in 75% yield. The structure of the product was substantiated by the ^1^HNMR (DMSO-*d_6_*) of compound **2a** revealing two singlet signal at δ 2.19 and 2.05 ppm which were readily assigned to the 12H attached at CH_3_, and a singlet at δ 4.55 (4H, s) assigned to the –NH_2_. The IR spectra of compound **2a** indicated a characteristic adsorption band at 3340–3217 cm^−1^ for the NH_2_ group.

Nevertheless, Phenylhydrazones **2b** were then synthesized in almost quantitative yields by the reaction between phenylhydrazine and **1**. The structure of phenylhydrazone derivatives was established on the basis of their elemental analysis and spectral data.

Microwave (MW) irradiation has been widely exploited in recent decades to carry out a striking number of organic syntheses, benefiting from dielectric heating in terms of reduced reaction times and increased yields, especially when coupled with solvent-free techniques. One of the most fertile applications in this field, also known as Microwave Assisted Organic Syntheses (MAOS) technique is heterocyclic chemistry, as reported in a recent review [23]. In particular, the synthetic pathways of pyrazole derivatives represent an interesting topic since these compounds have numerous applications in the pharmaceutical and agrochemical industry [24]. Some of the most widespread synthetic strategies to obtain these heterocyclic structures are the reactions between hydrazines with β-difunctional compounds [25] and 1,3-dipolar cycloaddition of diazo compounds onto multiple bonds [26].

**Scheme 2:**
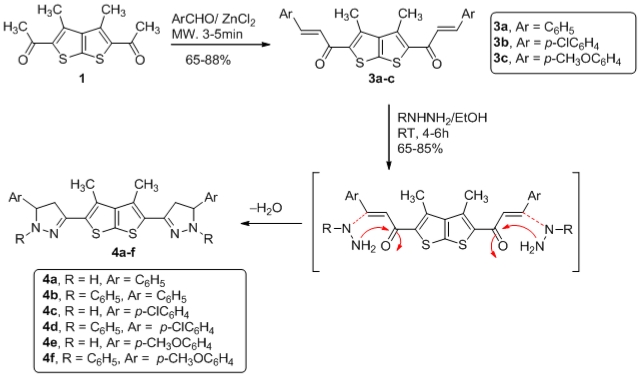
Synthesis of pyrazole derivatives **4a–f** from thienothiophene **1**.

Another approach which has been investigated would afford the pyrazole after an elimination/aromatization of the cycloadduct intermediate. On the basis of these studies in the present work, we utilized simple and mild solvent-free microwave mediated methodologies for the synthesis of pyrazoles derivatives from α,β-unsaturated carbonyl compounds. α,β-unsaturated carbonyl compounds **3a–c** were obtained starting from thieno [2,3-*b*] thiophene **1** coupled with corresponding aldehyde under microwave activation in the presence of ZnCl_2_. Pyrazoles derivatives **4a–f** were prepared following the classical procedure (ketone plus hydrazine derivatives in ethanol at reflux in very good yield as depicted in Scheme 2. The novel bis pyrazoles **4a–f** were assumed to be formed via a stepwise formation of hydrazone followed by a Michael 1,4-addition of the nucleophile nitrogen atom. We then decided to investigate the generality of this strategy and focused on the preparation of thieno[2,3-*b*]thiophenes pyrazole derivatives **4a–f**.

**Scheme 3:**
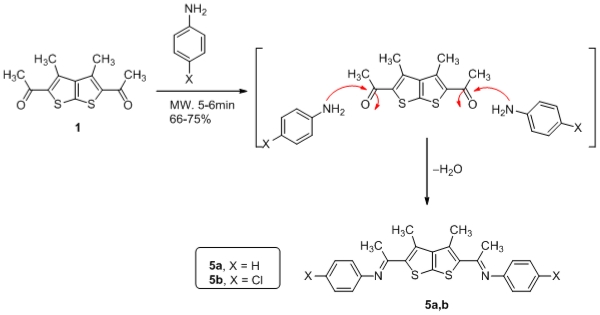
Synthesis of schiff’s base derivatives **5a,b** from thienothiophene **1**.

The utility of thieno[2,3-*b*]thiophenes **1** in the synthesis of schiff’s base bis-heterocycles **5a,b** was further explored via its reaction with aniline derivatives under microwave activation in the presence of ZnCl_2_. It is assumed that the product **5a,b** was formed via initial formation of nonisolable phenylamino ethanol derivatives followed by elimination of water molecules to give the desired product **5a,b** as drawn in Scheme 3. Spectral data (IR, NMR, MS) and elemental analysis was consistent with isolated product **5a,b**.

**Scheme 4:**
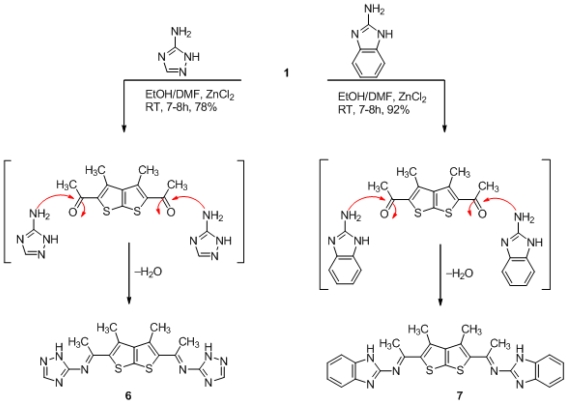
Synthesis of schiff’s base derivatives **6**, **7** from thienothiophene **1**.

On the other hand, the study was extended to investigate the behavoir of thieno[2,3-*b*]thiophenes derivatives **1** with different *N*-nucleophile like 1*H*-benzo[*d*]imidazol-2-amine and 1*H*-1,2,4-triazol-5-amine with a view to synthesizing various schiff’s base bis-heterocycles ring system. Thus, the reaction of **1** with these compounds in refluxing ethanol, in the presence of catalytic amount of ZnCl_2_, furnished the corresponding products **6** and **7** in very good to excellent yields Scheme 4.

## 3. Experimental Section

All melting points were measured on a Gallenkamp melting point apparatus in open glass capillaries and are uncorrected. IR spectra were measured as KBr pellets on a perking elmer FT 1000 spectrophotometer. The NMR spectra were recorded on a Varian Mercury Jeol-400 NMR spectrometer. ^1^H-NMR (400 MHz) and ^13^C-NMR (100 MHz) were run in deuterated dimethylsulphoxide (DMSO-*d_6_*). Chemical shifts (δ) are referred in terms of ppm and *J* -coupling constants are given in Hz. Abbreviations for multiplicity are as follows: s (singulet), d (doublet), t (triplet), q (quadruplet), m (multiplet). Mass spectra were recorded on a Shimadzu GCMS-QP 1000 EX mass spectrometer at 70 eV (EI). Elemental analysis was carried out on an Elementar Vario EL analyzer.

### 3.1. General Method for Preparation of Compounds Derivatives **2a,b** (GP1)

A mixture of Compound **1** (0.25 g, 1 mmol) with hydrazine derivatives (2 mmol) in absolute ethanol (20 mL, 99.9%) was refluxed for 4 h in the presence of TEA (triethyl amine). The reaction mixture was left to cool to RT. The formed solid product was filtered off and recrystallized from EtOH/DMF to afforded the corresponding hydrazones **2a,b**.

#### (3,4-dimethylthieno[2,3-b]thiophene-2,5-diyl)bis(ethan-1-yl-1-ylidene) bis (hydrazine) (**2a**)

Compound **2a** was prepared from hydrazine followed GP1 as yellow light scales crystals; Yield (95%); m.p.: 181 °C; IR (KBr) νmax 3340–3217 (NH_2_), 1625 (C=N) cm^−1; 1^H-NMR: δ 2.19, 2.05 (s, 12H, CH_3_), 4.55 (s, 4H, NH_2_); ^13^C-NMR: δ 15.0, 16.1, 126.8, 132.6, 140.1, 141.7, 157.5; MS *m/z* (%): 282 (M^+2^, 46), 280 (M^+^, 87), 266 (18), 248 (51); Anal. for C_12_H_16_N_4_S_2_ (280.41) calcd; C, 51.40; H, 5.75; N, 19.98; S, 22.87. Found: C, 51.10; H, 5.45; N, 19.68; S, 22.57.

#### (3,4-dimethylthieno[2,3-b]thiophene-2,5-diyl)bis(ethan-1-yl-1-ylidene)bis(1-phenylhydrazine) (**2b**)

Compound **2b** was prepared from phenylhydrazine followed GP1 as yellow powder crystals; Yield (75%); m.p.: 194 °C; IR (KBr) νmax 3202 (NH), 1580 (C=N) cm^−1; 1^H-NMR: δ 2.28, 2.19 (s, 12H, 2 CH_3_), 6.91–7.35 (m, 10H, ArH’s), 12.2 (br, 2H, NH); ^13^C-NMR: δ 14.8, 17.2, 113.2, 122.4, 123.2, 123.9, 127.2, 135.8, 140.7, 142.5, 157.5; MS *m/z* (%): 434 (M^+^, 17), 432 (M^+^, 35), 418 (56), 404 (25); Anal. for C_24_H_24_N_4_S_2_ (432.60) calcd; C, 66.63; H, 5.59; N, 12.95; S, 14.82. Found: C, 66.33; H, 5.89; N, 12.65; S, 14.52.

### 3.2. General Method for Preparation of Compounds Derivatives **3a**–**3c** (GP2)

A mixture of Compound **1** (0.25 g, 1 mmol) and aromatic aldhyde (2 mmol) with the addition of zinc chloride as a catalyst, was exposed to microwave irradiation at for 3–5 min. The formed solid product was filtered off and recrystallized from ethanol afforded the corresponding derivatives **3a–c**.

#### 1,1′-(3,4-dimethylthieno[2,3-b]thiophene-2,5-diyl)bis(3-phenylprop-2-en-1-one) (**3a**)

Compound **3a** was prepared from benzaldehyde followed GP2 as dark yellow powder crystals; Yield (75%); m.p.: 210 °C; IR (KBr) νmax: 1651 (C=O), 1573 (C=C) cm^−1; 1^H-NMR: δ 2.26 (s, 6H, CH_3_), 8.24 (d, 2H, *J* = 12.6 Hz, ethylene), 8.81 (d, 2H, *J* = 12.6 Hz, ethylene), 7.46–7.50 (m, 12H, ArH’s); ^13^C-NMR: δ 15.6, 113.3, 145.0, 126.2, 129.8, 129.9, 133.7, 138.6, 141.5, 147.7, 148.2, 186.6; MS *m/z* (%): 428 (M^+^, 80), 427 (M^+^, 75), 413 (20), 274(100); Anal. for C_26_H_20_O_2_S_2_ (428.57) calcd; C, 72.87; H, 4.70; O, 7.47; S, 14.96. Found: C, 72.57; H, 4.40; O, 7.77; S, 14.66.

#### 1,1′-(3,4-dimethylthieno[2,3-b]thiophene-2,5-diyl)bis(3-(4-chlorophenyl)prop-2- en-1-one) (**3b**)

Compound **3b** was prepared from *p*-chlorobenzaldehyde followed GP2 as greenish yellow cubes crystals; Yield (88%); m.p.: 265 °C; IR (KBr) νmax: 1721 (C=O), 1580 (C=C) cm^−1; 1^H-NMR: δ 2.33 (s, 6H, CH_3_), 7.70 (d, 2H, *J* = 12.4 Hz, ethylene), 8.47 (d, 2H, *J* = 12.6 Hz, ethylene), 7.93 (d, 2H, *J* = 8.8 Hz, ArH’s), 7.80(d, 2H, *J* = 8.8 Hz, ArH’s); ^13^C-NMR: δ 14.8, 112.6, 145.4, 125.8, 129.2, 131.1, 134.8, 137.1, 142.3, 148.3, 148.8, 185.8; MS *m/z* (%):498 (M^+^, 14), 496 (M^+^, 26), 495 (58), 426 (8); Anal. for C_26_H_18_Cl_2_O_2_S_2_ (497.46) calcd; C, 62.78; H, 3.65; Cl, 14.25; O, 6.43; S, 12.89. Found: C, 62.48; H, 3.35; Cl, 14.55; O, 6.13; S, 12.59.

#### 1,1′-(3,4-dimethylthieno[2,3-b]thiophene-2,5-diyl)bis(3-(4-methoxyphenyl)prop-2-en-1-one) (**3c**)

Compound **3c** was prepared from *p*-methoxybenzaldehyde followed GP2 as willowish white needles crystals; Yield (65%); m.p.: 239 °C; IR (KBr) νmax: 1732 (C=O), 1596 (C=C) cm^−1; 1^H-NMR: δ 2.29 (s, 6H, CH_3_), 3.49 (s, 6H, OCH_3_), 8.16 (d, 2H, *J* = 12.2 Hz, ethylene), 8.80 (d, 2H, *J* = 12.8 Hz, ethylene), 7.55 (d, 2H, *J* = 8.8 Hz, ArH’s), 7.97 (d, 2H, *J* = 8.8Hz, ArH’s); ^13^C-NMR: δ 16.2, 113.9, 145.6, 126.5, 128.8, 128.9, 136.0, 138.7, 140.7, 147.1, 147.8, 186.1; MS *m/z* (%): 488 (M^+^, 37), 487 (M^+^, 18), 474 (7), 457 (86); Anal. for C_28_H_24_O_4_S_2_ (488.62) calcd; C, 68.83; H, 4.95; S, 13.12 Found: C, 68.83; H, 4.65; S, 13.42.

### 3.3. General Method for Preparation of Compounds Derivatives **4a–f** (GP3)

To a mixture of 1,1′-(3,4-dimethylthieno[2,3-*b*]thiophene-2,5-diyl)bis(3-arylprop-2-en-1-one) derivatives (**3a–c**) (1 mmol) with hydrazine or phenylhydrazine (2 mmol) in mixture absolute ethanol (20 mL, 99.9%) and DMF (2–3 mL) was refluxed for 4–6 h. the reaction mixture was left to cool to RT. The formed solid product was filtered off and recrystallized from EtOH/DMF to afford the corresponding derivatives **4a–f**.

#### 3,3′-(3,4-dimethylthieno[2,3-b]thiophene-2,5-diyl)bis (5-phenyl-4,5-dihydro-1H-pyrazole) (**4a**)

Compound **4a** was prepared according to GP3 as yellow needles crystals; Yield (74%); m.p.: 270 °C; IR (KBr) νmax: 3250 (NH), 1624 (C=N) cm^−1; 1^H-NMR: δ 2.30 (s, 6H, CH_3_), 3.04 (dd, 4H, *J* = 10.5 Hz, *J* = 14.6 Hz, Methylene), 3.77 (dd, 4H, *J* = 10.5 Hz, *J* = 16.4 Hz, Methylene), 4.85 (dd, 4H, *J* = 10.2 Hz, *J* = 16.6 Hz, Methine), 7.27–7.39 (m, 12H, ArH’s), 8.84 (br, 2H, NH); ^13^C-NMR: δ 14.8, 51.3, 64.0, 121.9, 125.8, 126.0, 127.3, 138.6, 141.5, 147.8,148.4, 162.0; MS *m/z* (%): 456 (M^+^, 6), 454 (M^+^, 11), 441 (22), 240 (31); Anal. for C_26_H_24_N_4_S_2_ (456.63) calcd; C, 68.39; H, 5.30; N, 12.27; S, 14.04 Found: C, 68.09; H, 5.60; N, 11.97; S, 13.74.

#### 3,3′-(3,4-dimethylthieno[2,3-b]thiophene-2,5-diyl)bis(1,5-diphenyl-4,5-dihydro-1H-pyrazole) (**4b**)

Compound **4b** was prepared according to GP3 as light brown powder; Yield (65%); m.p.: 300 °C; IR (KBr) νmax: 1585 (C=N) cm^−1; 1^H-NMR: δ 2.32 (s, 6H, CH_3_), 3.13 (dd, 4H, *J* = 10.6 Hz, *J* = 14.1 Hz, Methylene), 3.76 (dd, 4H, *J* = 10.2 Hz, *J* = 16.1 Hz, Methylene), 4.88 (dd, 4H, *J* = 10.6 Hz, *J* = 16.2 Hz, Methine), 7.22–7.50 (m, 24H, ArH’s); ^13^C-NMR: δ 14.8, 51.6, 65.2, 121.8, 122.2, 124.7, 125.8, 126.8, 128.2, 129.3, 131.8, 137.3, 140.5, 142.2, 144.7, 161.2; MS *m/z* (%): 608 (M^+^, 17), 606 (M^+^, 67), 593 (13), 531(58); Anal. for C_38_H_32_N_4_S_2_ (608.82) calcd; C, 74.97; H, 5.30; N, 9.20; S, 10.53 Found: C, 74.67; H, 5.00; N, 9.50; S, 10.23.

#### 3,3′-(3,4-dimethylthieno[2,3-b]thiophene-2,5-diyl)bis(5-(4-chlorophenyl)-4,5-dihydro-1H-pyrazole) (**4c**)

Compound **4c** was prepared according to GP3 as yellowish white powder; Yield (75%); m.p.: 320 °C; IR (KBr) νmax: 3298 (NH), 1620 (C=N) cm^−1; 1^H-NMR: δ 2.35 (s, 6H, CH_3_), 3.04 (dd, 4H, *J* = 10.6 Hz, *J* = 14.1 Hz, Methylene), 3.77 (dd, 4H, *J* = 10.2 Hz, *J* = 16.1 Hz, Methylene), 4.81 (dd, 4H, *J* = 10.66 Hz, *J* = 16.22 Hz, Methine), 7.90 (d, 2H, *J* = 8.8 Hz, ArH’s), 8.30 (d, 2H, *J* = 8.8 Hz, ArH’s), 8.87 (br, 2H, NH); ^13^C-NMR: δ 15.4, 52.2, 64.3, 126.6,128.7, 131.1, 132.4, 136.6, 142.0, 142.9, 146.6, 158.8; MS *m/z* (%): 526 (M^+^, 61), 524 (M^+^, 82), 522 (17), 509(26); Anal. for C_26_H_22_Cl_2_N_4_S_2_ (525.52) calcd; C, 59.42; H, 4.22; Cl, 13.49; N, 10.66; S, 12.20. Found: C, 59.12; H, 4.52; Cl, 13.19; N, 10.36; S, 12.50.

#### 3,3′-(3,4-dimethylthieno[2,3-b]thiophene-2,5-diyl)bis(5-(4-chlorophenyl)-1-phenyl-4,5-dihydro-1Hpyrazole) (**4d**)

Compound **4d** was prepared according to GP3 as brown cubes Crystals; Yield (67%); m.p.: 320 °C; IR (KBr) νmax: 1584 (C=N) cm^−1; 1^H-NMR: δ 2.31 (s, 6H, CH_3_), 3.23 (dd, 4H, *J* = 10.6 Hz, *J* = 14.1 Hz, Methylene), 3.79 (dd, 4H, *J* = 10.2 Hz, *J* = 16.1 Hz, Methylene), 4.83 (dd, 4H, *J* = 10.5 Hz, *J* = 16.2 Hz, Methine), 7.24–7.29 (m, 10H, phenyl), 7.92 (d, 2H, *J* = 8.8 Hz, ArH’s), 8.00 (d, 2H, *J* = 8.8 Hz, ArH’s); ^13^C-NMR: δ 15.6, 50.8, 63.8, 120.3, 122.6, 124.8, 125.4, 125.9, 127.1, 130.6, 133.5, 135.6, 143.3, 143.9, 145.2, 158.8; MS *m/z* (%): 678 (M^+^, 13), 676 (M^+^, 78), 661 (9), 641 (84); Anal. for C_38_H_30_Cl_2_N_4_S_2_ (677.71) calcd; C, 67.35; H, 4.46; Cl, 10.46; N, 8.27; S, 9.46. Found: C, 67.05; H, 4.16; Cl, 10.16; N, 8.57; S, 9.16.

#### 3,3′-(3,4-dimethylthieno[2,3-b]thiophene-2,5-diyl)bis(5-(4-methoxyphenyl)-4,5-dihydro-1Hpyrazole)( **4e**)

Compound **4e** was prepared according to GP3 as yellow powder crystals; Yield (77%); m.p.: 295 °C; IR (KBr) νmax: 3272 (NH), 1582 (C=N) cm^−1; 1^H-NMR: δ 2.34 (s, 6H, CH_3_), 3.48 (s, 6H, Methoxy), 3.75 (dd, 4H, *J* = 10.6 Hz, *J* = 14.1 Hz, Methylene), 3.80 (dd, 4H, *J* = 10.5 Hz, *J* = 16.4 Hz, Methylene), 4.85 (dd, 4H, *J* = 10.5 Hz, *J* = 16.2 Hz, Methine), 7.56 (d, 2H, *J* = 8.8 Hz, ArH’s), 7.95 (d, 2H, *J* = 8.8 Hz, ArH’s), 8.90 (br, 2H, NH); ^13^C-NMR: δ 14.7, 41.6, 53.1, 65.6, 126.1, 127.9, 131.4, 134.2, 134.5, 146.0, 148.2, 148.6, 161.9; MS *m/z* (%): 516 (M^+^, 88), 514 (M^+^, 56), 501 (62), 485 (9); Anal. for C_28_H_28_N_4_O_2_S_2_ (516.68) calcd; C, 65.09; H, 5.46; N, 10.84; S, 12.41. Found: C, 64.79; H, 5.16; N, 10.54; S, 12.11.

#### 3,3′-(3,4-dimethylthieno[2,3-b]thiophene-2,5-diyl)bis(5-(4-methoxyphenyl)-1-phenyl-4,5-dihydro-1Hpyrazole)( **4f**)

Compound **4f** was prepared according to GP3 as dark yellow powder crystals; Yield (85%); m.p.: 300 °C; IR (KBr) νmax: 1622 (C=N) cm^−1; 1^H-NMR: δ 2.28 (s, 6H, CH_3_), 3.47(s, 6H, Methoxy), 3.02 (dd, 4H, *J* = 10.6 Hz, *J* = 14.6 Hz, Mthylene), 3.76 (dd, 4H, *J* = 10.5 Hz, *J* = 16.4 Hz, Mthylene), 4.87 (dd, 4H, *J* = 10.5 Hz, *J* = 16.2 Hz, Methine), 7.22–7.28 (m, 10H, phenyl), 7.58 (d, 2H, *J* = 8.8 Hz, ArH’s), 7.96 (d, 2H, *J* = 8.8 Hz, ArH’s); ^13^C-NMR: δ 14.6, 42.6, 55.1, 66.0, 119.86, 122.74, 124.3, 126.1, 126.2, 128.1, 131.1, 133.6, 135.9, 145.7, 147.2, 148.4, 162.3; MS *m/z* (%):668 (M^+^, 88), 666 (M^+^, 16), 653 (38), 638 (58); Anal. for C_40_H_36_N_4_O_2_S_2_ (516.68) calcd; C, 71.83; H, 5.42; N, 8.38; S, 9.59. Found: C, 71.53; H, 5.22; N, 8.08; S, 9.89.

### 3.4. General Method for Preparation of Compounds Derivatives **5a,b** (GP4)

A mixture of Compound **1** (0.25 g, 1 mmol) with aniline derivatives (2 mmol) with the addition of zinc chloride as a catalyst, was exposed to microwave irradiation at for 5–6 min. The formed solid product was filtered off and recrystallized from ethanol afforded the corresponding derivatives **3a–c**. *(3,4-dimethylthieno[2,3-*b*]thiophene-2,5-diyl)bis(ethan-1-yl-1-ylidene) aniline derivatives (****5a****).* Compound **5a** was prepared from aniline followed GP4 as white scales crystals; Yield (75%); m.p.: 260 °C; IR (KBr) νmax: 1600 (C=N) cm^−1; 1^H-NMR: δ 2.33, 2.05 (s, 12H, CH_3_), 6.48–6.99 (m, 10H, aromatic); ^13^C-NMR: δ 15.6, 24.07, 122.0, 126.0, 126.9, 127.6, 138.5, 141.8, 148.0, 148.3, 166.0; MS *m/z* (%): 402 (M^+^, 98), 400 (M^+^, 42), 387 (76), 248 (58); Anal. for C_24_H_22_N_2_S_2_ (402.57) calcd; C, 71.60; H, 5.51; N, 6.96; S, 15.93. Found: C, 71.30; H, 5.21; N, 6.66; S, 15.63.

#### (3,4-dimethylthieno[2,3-b]thiophene-2,5-diyl)bis(ethan-1-yl-1-ylidene)bis(4-chloroaniline)(**5b**)

Compound **5b** was prepared from *p*-chloroaniline followed GP4 as yellowish white powder Crystals; Yield (66%); m.p.: 230 °C; IR (KBr) νmax: 1575 (C=N) cm^−1; 1^H-NMR: δ 2.25, 2.12 (s, 12H, CH_3_), 6.50 (d, *J* = 7.2 Hz, 2H, ArH’s), 6.72 (d, *J* =7.2 Hz, 2H, ArH’s); ^13^C-NMR: δ 14.8, 23.8, 125.3, 127.4, 128.2, 131.1, 137.2, 139.1, 146.4, 147.8, 164.5; MS *m/z* (%):472 (M^+^, 56), 470 (M^+^, 48), 468 (9), 435 (18); Anal. for C_24_H_20_Cl_2_N_2_S_2_ (471.46) calcd; C, 61.14; H, 4.28; Cl, 15.04; N, 5.94; S, 13.60. Found: C, 61.44; H, 3.98; Cl, 14.74; N, 5.64; S, 13.30.

#### (3,4-dimethylthieno[2,3-b]thiophene-2,5-diyl)bis(ethan-1-yl-1-ylidene)bis(1H-1,2,4-triazol-5-amine) (**6**)

A mixture of Compound **1** (0.25 g, 1 mmol) with amino triazole (0.16 mL, 2 mmol) in absolute ethanol (20 mL, 99.9%) with addition of a little DMF (2 mL) was refluxed for 7–8 h. then left to cool. The formed solid product was filtered off and recrystallized from EtOH/DMF to afforded the corresponding compound **6** as dark yellow powder Crystals; Yield (78%); m.p.: 256 °C; IR (KBr) νmax 3332 (NH), 1620 (C=N) cm^−1; 1^H-NMR: δ 2.10, 2.64 (s, 12H, CH_3_), 7.86 (s, 2H, triazole), 13.02 (s, 2H, NH); ^13^C-NMR: δ 14.2, 20.2, 145.0, 155.2, 135.9, 141.5, 147.8, 148.2, 162.3; MS *m/z* (%): 384 (M^+^, 83), 382 (M^+^, 15), 369 (26), 275 (46); Anal. for C_16_H_16_N_8_S_2_ (384.48) calcd; C, 49.98; H, 4.19; N, 29.14; S, 16.68 Found: C, 49.68; H, 4.49; N, 28.84; S, 16.38.

#### (3,4-dimethylthieno[2,3-b]thiophene-2,5-diyl)bis(ethan-1-yl-1-ylidene)bis(1H-benzo[d]imidazol-2- amine) (**7**)

A mixture of Compound **1** (0.25 g, 1 mmol) with amino benzoimidazol (0.26 mL, 2 mmol) in absolute ethanol (20 mL, 99.9%) with addition a little of DMF (2 mL) was refluxed for 7–8 h. then left to cool. The formed solid product was filtered off and recrystallized from EtOH/DMF to afforded the corresponding compound **7** as greenish yellow powder Crystals; Yield (92%); m.p.: 320 °C; IR (KBr) νmax: 3331 (NH), 1600 (C=N) cm^−1; 1^H-NMR: δ 1.23, 2.82 (s, 12H, CH_3_), 7.15–7.35 (m, 8H, Benzoimidazol), 7.74 (s, 2H, NH); ^13^C-NMR: δ 14.9, 19.0, 102.3, 120.5, 123.1, 126.9, 128.9, 134.1, 135.9, 144.5, 148.2, 160.0; MS *m/z* (%): 482 (M^+^, 42), 480 (M^+^, 78), 467 (100), 451 (24); Anal. for C_26_H_22_N_6_S_2_ (384.48) calcd; C, 64.70; H, 4.59; N, 17.41; S, 13.29 Found C, 64.40; H, 4.29; N, 17.11; S, 13.59.

## 4. Conclusions

In summary, we have successfully prepared various polysubstituted thieno[2,3-*b*]thiophenes based on the reaction of carbonyl compounds with appropriate *N*-nucleophile assisted by microwave or classical methods. The simple procedure, mild conditions, high yields and especially environmental friendliness make this protocol very attractive, and may possess interesting biological properties or applications in material sciences.

## References

[1] Litvinov V.P., Gol’dfarb Y.L., Katritzky A.R., Boulton A.J. (1976). Advanced Heterocyclic Chemistry.

[2] King W.J., Nord F.F. (1949). Studies in the thiophene series. V. Wolff-Kishner reductions. J. Org. Chem.

[3] Wu C., Decker E.R., Blok N., Bui H., You T.J., Wang J., Bourgoyne A.R., Knowles V., Berens K.L., Holland G.W., Brock T.A., Dixon R.A.F. (2004). Discovery, modeling, and human pharmacokinetics of *N*-(2-acetyl-4,6-dimethylphenyl)-3-(3,4-dimethylisoxazol-5-ylsulfamoyl) thiophene-2-carboxamide (TBC3711), a second generation, ET_A_ selective, and orally bioavailable endothelin antagonist. J. Med. Chem.

[4] Doré K., Dubus S., Ho H.A., Lévesque I., Brunette M., Corbeil G., Boissinot M., Boivin G., Bergeron M.G., Boudreau D., Leclerc M. (2004). Fluorescent polymeric transducer for the rapid, simple, and specific detection of nucleic acids at the zeptomole level. J. Am. Chem. Soc.

[5] Rost C., Karg S., Riess W., Loi M.A., Murgia M., Muccini M. (2004). Ambipolar light-emitting organic field-effect transistor. Appl. Phys. Lett.

[6] Vriezema D.M., Hoogboom J., Velonia K., Takazawa K., Christianen P.C.M., Maan J.C., Rowan A.E., Nolte R.J.M. (2003). Vesicles and polymerized vesicles from thiophene-containing rod-coil block copolymers. Angew. Chem. Int. Ed.

[7] Yu H., Pullen A.E., Büchel M.G., Swager T.M. (2004). Charge-specific interactions in segmented conducting polymers: An approach to selective ionoresistive responses. Angew. Chem. Int. Ed.

[8] Mabkhot Y.N., Kheder N.A., Al-Majid A.M. (2010). Facile and convenient synthesis of new thieno[2,3-*b*]thiophene derivatives. Molecules.

[9] Mabkhot Y.N. (2010). synthesis and chemical characterisation of new bis-thieno[2,3-*b*]thiophene derivatives. Molecules.

[10] Mabkhot Y.N. (2009). Synthesis and analysis of some bis-heterocyclic compounds containing Sulphur. Molecules.

[11] Sabir H.M., Sangvikar Y.S., Ghadigaonkar S.G., Ashraf M., Meetsma A. (2008). Oxa-bridged cyclophanes featuring thieno[2,3-*b*]thiophene and *C**_2_*-symmetric binol or bis-naphthol rings: Synthesis, structures, and conformational studies. Tetrahedron.

[12] Sabir H.M., Ashraf M., Hariharasubrahmanian H., Kelloggb R.M., Meetsma A. (2004). Donor-acceptor thieno[2,3-*b*]thiophene systems: Synthesis and structural study of 3-anisyl-4-pyridyl(pyridinium) thieno[2,3-*b*]thiophenes. J. Mol. Struct..

[13] Bugge A. (1969). preparation of some brominated thieno[2,3-*b*]thiophenes and thieno[3,2-*b*]thiophenes. Acta Chem. Scand.

[14] Mashraqui S.H., Sangvikar Y.S., Ashraf M., Kumar S., Daub E. (2005). Dipyridyl/pyridinium thieno[2,3-*b*]thiophenes as new atropisomeric systems. Synthesis, conformat-ional analysis and energy minimization. Tetrahedron.

[15] Liu M.-G., Hu Y.-G., Ding M.-W. (2008). New iminophosphorane-mediated synthesis of thieno[3′,2′:4,5]thieno[3,2-*d*]pyrimidin-4(3*H*)-ones and 5*H*-2,3-dithia-5,7-diaza-cyclopenta[*c*,*d*] indenes. Tetrahedron.

[16] Wu Y.X., Cao J., Deng H.Y., Feng J.X. (2011). Synthesis, complexation, and fluorescence behavior of 3,4-dimethylthieno[2,3-*b*]thiophene carrying two monoaza-15-crown-5 ether groups. Spectrochim. Acta Part A.

[17] McCulloch I., Heeney M., Chabinyc M.L., DeLongchamp D., Kline R.J., Cölle M., Duffy W., Fischer D., Gundlach D., Hamadani B. (2009). Semiconducting thienothiophene copolymers: Design, synthesis, morphology, and performance in thin-film organic transistors. Adv. Mater.

[18] Sabir H.M., Sanghvikar Y., Ghadhigaonkar S., Kumar S., Meetsma A., Trân Huu Dâu E. (2009). [3.3]Dithia-bridged cyclophanes featuring a thienothiophene ring: Synthesis, structures and conformational analysis. Beilstein. J. Org. Chem..

[19] Heeney M., Bailey C., Genevicius K., Shkunov M., Sparrowe D., Tierney S., McCulloch I. (2005). Stable polythiophene semiconductors incorporating Thieno[2,3-*b*]thiophene. J. Am. Chem. Soc.

[20] Egbertson M.S., Cook J.J., Bednar B., Prugh J.D., Bednar R.A., Gaul S.L., Gould R.J., Hartman G.D., Homnick C.F., Holahan M.A. (1999). Non-peptide GPIIb/IIIa inhibitors. 20. Centrally constrained thienothiophene α-sulfonamides are potent, long acting in *vivo* inhibitors of platelet aggregation. J. Med. Chem.

[21] Mabkhot Y.N., Al-Majid A.M., Assem B., Alshahrani S., Siddiqui Y. (2011). 1,1′-(3-Methyl-4- phenylthieno[2,3-*b*]thiophene-2,5-diyl)diethanone as building block in heterocyclic synthesis. Novel synthesis of some pyrazoles, and pyrimidines derivatives. Molecules.

[22] Mabkhot Y.N., Al-Majid A.M., Alamary A.S. (2011). Synthesis and chemical characterisation of some new diheteroaryl thienothiophene derivatives. Molecules.

[23] Bougrin K., Loupy A. (2005). Microwave-assisted solvent-free heterocyclic synthesis. J. Photochem. Photobiol. C Photochem. Rev.

[24] Aggarwal V.K., de Vicente J., Bonnert R.V. (2003). A novel one-pot method for the preparation of pyrazoles by 1,3-dipolar cycloadditions of diazo compounds generated in situ. J. Org. Chem.

[25] Kost A.N., Grandberg I.I. (1966). Progress in pyrazole chemistry. Adv. Heterocycl. Chem.

[26] Padwa A (1984). 1,3-Dipolar Cycloaddition Chemistry.

